# Improving the stratification power of cardiac ventricular shape

**DOI:** 10.1186/1532-429X-17-S1-O77

**Published:** 2015-02-03

**Authors:** Gerardo Gonzalez, Daniel Nolte, Adam J Lewandowski, Paul Leeson, Nicolas P Smith, Pablo Lamata

**Affiliations:** 1 Dept. Biomedical Engineering, King's College London, London, UK; 2 Dept. Cardiovascular Medicine, University of Oxford, Oxford, UK; 3 Faculty of Engineering, University of Auckland, Auckland, New Zealand

## Background

Cardiac MRI has become the reference imaging modality used to assess left ventricular (LV) remodelling. Ventricles are generally characterised by bulk metrics such as cavity volume, mass or wall thickness. We introduce a workflow that aims to improve the stratification power of conventional analysis by providing 3D shape metrics through the construction of computational cardiac atlases.

## Methods

Computational 3D meshes of the LV are personalised to MRI images, capturing and encoding shape information. The method requires the provision of segmented myocardial images, and a parameter to choose the relative importance between minimise noise and artefacts and maximise accuracy in the personalisation of meshes. Subsequently, the principal modes of anatomical variation are extracted from the set of 3D meshes that constitute the atlas. These principal modes represent how ventricular shape changes within the population under study. The anatomy of each subject is then described by a set of shape indexes that are used for further stratification and analysis. This fully automatic procedure has been made available through an online cloud infrastructure (http://www.vph-share.eu). Its use is illustrated by the analysis of a cohort of 224 subjects. Specifically, we report how the shape of the LV in adulthood (between 20 and 30 year-old individuals) can predict gestational age (either premature or term-birth), comparing the performance of traditional metrics (length, diameter, volume and mass) to the first three atlas-derived descriptors.

## Results

Ventricular meshes were fully automatically reconstructed from the end diastolic frame of cine-SSFP sequences, with average fitting error of 1.25mm. Gestational age was predicted with 85% specificity and 88% sensitivity based on traditional metrics, improving to 93% and 98% respectively with atlas-derived shape descriptors.

## Conclusions

Shape of the left ventricle described by a computational atlas has the potential to improve the stratification of disease processes. Researchers are invited and encouraged to use this tool that has been made available to the community.

## Funding

This work was partly supported by the FP7 VPH-Share project under Grant agreement n.269978. P.L. holds a Sir Henry Dale Fellowship jointly funded by the Wellcome Trust and the Royal Society, grant n.099973/Z/12/Z.

